# Group hypnosis vs. relaxation for smoking cessation in adults: a cluster-randomised controlled trial

**DOI:** 10.1186/1471-2458-13-1227

**Published:** 2013-12-23

**Authors:** Maria Dickson-Spillmann, Severin Haug, Michael P Schaub

**Affiliations:** 1 Swiss Research Institute for Public Health and Addiction ISGF, University of Zurich, Konradstrasse 32, Postfach, 8031 Zürich, Switzerland

**Keywords:** Smoking cessation, Cigarette smoking, Hypnosis, Group therapy, Cluster-randomised controlled trial

## Abstract

**Background:**

Despite the popularity of hypnotherapy for smoking cessation, the efficacy of this method is unclear. We aimed to investigate the efficacy of a single-session of group hypnotherapy for smoking cessation compared to relaxation in Swiss adult smokers.

**Methods:**

This was a cluster-randomised, parallel-group, controlled trial. A single session of hypnosis or relaxation for smoking cessation was delivered to groups of smokers (*median* size = 11). Participants were 223 smokers consuming ≥ 5 cigarettes per day, willing to quit and not using cessation aids (47.1% females, *M* = 37.5 years [*SD* = 11.8], 86.1% Swiss). Nicotine withdrawal, smoking abstinence self-efficacy, and adverse reactions were assessed at a 2-week follow-up. The main outcome, self-reported 30-day point prevalence of smoking abstinence, was assessed at a 6-month follow up. Abstinence was validated through salivary analysis. Secondary outcomes included number of cigarettes smoked per day, smoking abstinence self-efficacy, and nicotine withdrawal.

**Results:**

At the 6-month follow up, 14.7% in the hypnosis group and 17.8% in the relaxation group were abstinent. The intervention had no effect on smoking status (*p* = .73) or on the number of cigarettes smoked per day (*p* = .56). Smoking abstinence self-efficacy did not differ between the interventions (*p* = .14) at the 2-week follow-up, but non-smokers in the hypnosis group experienced reduced withdrawal (*p* = .02). Both interventions produced few adverse reactions (*p* = .81).

**Conclusions:**

A single session of group hypnotherapy does not appear to be more effective for smoking cessation than a group relaxation session.

**Trial registration:**

Current Controlled Trials ISRCTN72839675.

## Background

Many smokers would like to give up smoking. Studies indicate that between 40–80% are willing to quit [1-3]. Up to 80% try to quit smoking without using any assistance [4,5]. However, long-term success with the “cold turkey method” is low at 7–8% [5,6]. As smokers hold misperceptions regarding nicotine replacement therapies (NRT) for smoking cessation, these methods are under-used [7-11]. For those smokers who are looking for cessation assistance, but who are not motivated to try NRT or other medications for smoking cessation, a range of other cessation methods, including hypnotherapy, is available. At least one-quarter of smokers who intend to quit are interested in hypnotherapy [12,13]. There is also remarkable interest among Swiss smokers [14].

The efficacy of hypnosis as a smoking cessation method has been investigated [15-26], but there is heterogeneity in study designs. Several reviews and meta-analyses on hypnotherapy for smoking cessation have been undertaken [27-31] which, apart from two exceptions [32,33], have not been able to clearly support the efficacy of hypnotherapy as a smoking cessation method.

In this paper, we report findings from a cluster-randomised trial that investigates the efficacy of hypnotherapy for smoking cessation compared to relaxation. Our trial stands out from previous research as it is characterised by a large sample size, comparison is made with an active control group, and self-reported smoking abstinence is biologically validated. Our trial therefore represents a substantial addition to the relatively small and under-powered body of research on hypnosis for smoking cessation. We intend to evaluate hypnotherapy as an affordable, time-saving therapy for smokers with quit intentions. Therefore, the intervention in our trial is conducted in a single session, group format.

## Methods

### Trial design

The protocol for this study has been previously published [34]. Regarding the trial design, a divergence between the protocol and the present article should be noted. The protocol had announced a simple randomised trial. When recruitment for the study started, however, we became aware that according to our recruitment procedure, the unit of randomisation was clusters of individuals rather than individuals. This might result in participants from a particular cluster having more features in common with each other than with participants from other clusters (e.g. the case of work colleagues attending the therapy session together). Therefore, this was effectively a cluster-randomised, parallel-group, controlled trial. The clusters refer to the groups of participants who attended the therapy sessions together. Two weeks after the intervention, all the participants were contacted by telephone for a follow-up interview. Six months after the intervention, another follow-up interview was conducted by telephone.

The relaxation condition was intended to be an active control group (as opposed to a waiting list control) that matched the experimental group with regard to therapist contact time [29]. By choosing this type of control group, we wanted to evaluate whether hypnotherapy had an effect in addition to the non-specific effects of therapist contact, social support and relaxation. We did not include a waiting list control group as we anticipated that participants in this kind of control group would not feel any motivation to quit during the follow-up period, thus biasing six-month abstinence rates.

### Participants

Recruitment was performed through advertisements in online and print newspapers. Individuals over 18 years who reported smoking at least five cigarettes a day, not using any other cessation method at the time of the study, and intending to quit were eligible to participate. In contrast to the study protocol, we did not apply an upper age limit as one older individual (age 78) fulfilled all other inclusion criteria and we did not anticipate any methodological or ethical reasons to exclude this individual. Exclusion criteria were acute alcohol or substance use other than nicotine and manifest signs of psychotic symptoms as observed by the therapist at the start of the therapy sessions.

All interested individuals were mailed information about the study, in particular regarding inclusion criteria, cost of participation, anonymity, and confidentiality. Further, they were informed that the efficacy of hypnosis and relaxation in smoking cessation was unclear, that there were very low risks associated with these interventions, and that they would be randomly assigned to either intervention. The participants were asked not to use any other smoking cessation aids throughout the study period. To ensure their commitment to smoking cessation and study participation, participants paid 40 Swiss Francs (ca. 37 USD) in the course of the therapy session.

Potential participants were provided with a calendar showing all possible therapy dates. When a group of 8–15 eligible smokers indicated availability for the same date, the project leader assigned the group (cluster) to the next intervention in the sequence.

The therapy sessions took place in the conference rooms of hotels or the institutions involved, either in Zurich city or in a small town in Northwest Switzerland between 8–10 pm on weekdays and between 10–12 am on Saturdays.

### Interventions

The therapy sessions were conducted in Swiss German language by a 37-year old male hypnosis and relaxation therapist. The therapy sessions consisted of three parts: a psycho-educational part (40 minutes), the actual intervention (40 min), and a debriefing (20 min). The contents of each part were recorded in a script which the therapist was instructed to follow. The psycho-educational part, which consisted of discussing the benefits of smoking cessation, was equal in both conditions. In the second part, the actual intervention took place with dimmed lights and soft background music. Hypnosis was induced using guided imagery. Using a calm tone of voice, the therapist invited the participants to travel through their body and to progressively feel heavier, warmer and more comfortable. For example, the participants were asked to “feel your toes, heels, ankles, calves, knees and thighs becoming heavier and heavier”, to finally “experience the comfortable heaviness of your whole legs” and then to “sink deeper and deeper into this state”. This exercise was repeated until the whole body was covered. The induction of hypnosis required ten minutes (an extension of five minutes compared to the protocol) before the first set of suggestions was made to disconnect pleasant experiences, such as socialising, from the act of smoking. Hypnosis was then deepened by repeating statements involving relaxation and by associating (*“anchoring”*) the resulting state of deep relaxation with a key word that was subsequently repeated to maintain this state. During deep relaxation, the participants were given suggestions targeted at switching their self-image from that of smokers to non-smokers. The suggestions contained elements of cognitive-behavioural approaches to hypnotherapy for smoking cessation [21,35,36]. Suggestions were made for the participants to use their power to resist smoking in tempting situations and to deal with symptoms such as mood swings or enhanced appetite following smoking cessation. Another set of suggestions referred to evoking a positive commitment to smoking cessation, assuming responsibility for the own body and reducing the physiological and psychological effects of smoking withdrawal [16,17,24,37]. At the end of the session, the participants were led back to full awareness.

In the relaxation condition, the participants were initially invited to make themselves comfortable and to relax. No repetitive statements were made, and no anchors were used to reinforce and deepen relaxation. The participants were asked to listen to the music for ten minutes before the same suggestive sentences as used in the hypnosis group were given.

All participants were debriefed about the intervention at the end of the session. The debriefing included information on the intervention they had undergone and responding any open questions from the participants. Furthermore, the participants received a compact disc (CD) for use at home that included the contents of the actual intervention.

To ensure fidelity to the intervention script, the project leader performed several random, unannounced visits to the therapy sessions. There was no indication of any breach of the script by the therapist.

### Outcomes

The primary outcome of this trial was the rate of smoking abstinence six months following hypnotherapy, compared to relaxation. As secondary outcomes, we compared nicotine withdrawal symptoms in ex-smokers between the interventions, and we evaluated intervention differences in self-efficacy and adverse reactions two weeks after the interventions. Furthermore, we compared daily cigarette consumption in non-quitters between the interventions two weeks and six months after the therapy sessions.

Before the first part of the therapy session, the participants filled in a range of baseline questionnaires. These questionnaires assessed sociodemographic information, nicotine dependence via the Fagerström Test for Nicotine Dependence (FTND) [38,39]; smoking history; smoking abstinence self-efficacy [40,41]; history of other substance use; mental health via the Beck Depression Inventory-V (BDI-V) [42,43], the Beck Anxiety Inventory (BAI) [44,45], and the mental component score (MCS-12) of the 12-Item Short Form Health Survey (SF-12) [46,47]; and physical health via body mass index (BMI) and the physical component score (PCS-12) of the SF-12 [46,47]. Lifetime diagnoses of anxiety, depression, lung disease, cancer, and heart disease were assessed [48]. In addition, the participants provided a baseline saliva sample (Quantisal® saliva collection device, nal von minden, Regensburg, Germany). This sample was used to determine the concentration of cotinine, which is a metabolite of nicotine, via liquid chromatography-mass spectrometry/mass spectrometry (LC-MS/MS) analysis at the Institute of Forensic Medicine of the University of Zurich.

In the two-week follow-up interview, the seven-day point prevalence smoking abstinence, frequency of use of the CD since the therapy session, smoking abstinence self-efficacy, withdrawal symptoms through the Minnesota Nicotine Withdrawal scale (MNWS) [49], potential adverse events resulting from the intervention, BAI, and BDI-V were assessed. The adverse events included common symptoms that are not typically associated with nicotine withdrawal. These symptoms were assessed on 4-point scales ranging from 1 (*not present*) to 4 (*severe*).

At the six-month follow-up interview, the 30-day point prevalence smoking abstinence, use of the CD in the previous 30 days, use of other cessation aids, BAI, BDI-V, and adverse events were investigated. Those participants who reported smoking abstinence for the previous 30 days and who had not used other cessation aids were sent a saliva collection device by post.

Results on the BMI at baseline, the BAI and BDI-V at both follow-ups, and adverse events at the six-month follow-up are not reported in the present paper.

### Sample size

The cluster-randomized design of this trial required a recalculation of the sample size compared to the study protocol [34]. We recalculated sample size on the basis of group hypnosis studies that did not use nicotine replacement therapy. In the absence of more recent investigations we referred to three early trials of group hypnotherapy and smoking cessation [18,26,50]. Of these, we used the most conservative result of 25% abstinence after nine months [18]. That study observed 0% abstinence in the attention-placebo control group. However, we assumed a rate of cessation without any aids of 7% [6] for the control group. We aimed for a statistical power of 80% and accepted an alpha level of 5%. In an individual trial, the target sample size for the current trial would have been 142 individuals (G*Power, University of Kiel, Germany). We assumed an *ICC* of 0.05 and an average cluster size of 10 individuals. Thus, the design effect was *D =* 1 + (10–1) × 0.05 = 1.45 [51]. Therefore, the required minimum sample size in the present trial was *N =* 142 × 1.45 = 206. Our actual sample size was 257.

### Implementation

#### Sequence generation

Assuming an average of 10 individuals per group, the project leader generated a random sequence of 20 sessions through an online program, with the criterion that the occurrence of both interventions had to be balanced (i.e., 10 sessions per intervention). When more participants signed up for the trial, one last session was randomly allocated by the online program to the hypnosis intervention.

### Ethical approval and informed consent

This cluster-randomised trial was performed in compliance with the Declaration of Helsinki and has been reviewed by the Ethics Committee of the Canton of Zurich, which did not declare any objections (KEK-StV-Nr.16/10). Participants were sent an informed consent form prior to the therapy session. They were asked to take the signed form with them to the therapy session.

### Blinding

The hypnosis and relaxation therapist was informed via text message by the project leader after the first part of the therapy session about which intervention he was about to perform. The participants remained blind with regard to their assigned intervention until the end of the therapy session.

### Statistical methods

Chi-square and t-tests were used to compare baseline characteristics between the hypnosis and the relaxation group. The data analysis was conducted according to the intention-to-treat (ITT) principle. In parallel, we analysed our data on a complete-case basis. When analyses were performed in a subsample, we re-checked for baseline differences. The primary outcome was analysed using logistic regression analysis with “hypnotherapy vs. relaxation” as the independent variable, “smoking yes/no” as the dependent variable, and variables with significant baseline differences as covariates. Secondary outcomes were analysed using linear and ordinal regression analysis. We did not use generalised estimating equations (GEE) as originally intended [34] since this procedure was less suitable to the present outcomes than logistic regression analysis. STATA 12 SE (College Station, Texas, USA) was used for the regression analyses. To account for cluster-randomization, the survey (svy) command was used in the calculation of all outcomes. This command accurately estimates standard errors when the sampling method is other than simple random sampling.

## Results

### Flow of the participants

**Figure 1:**
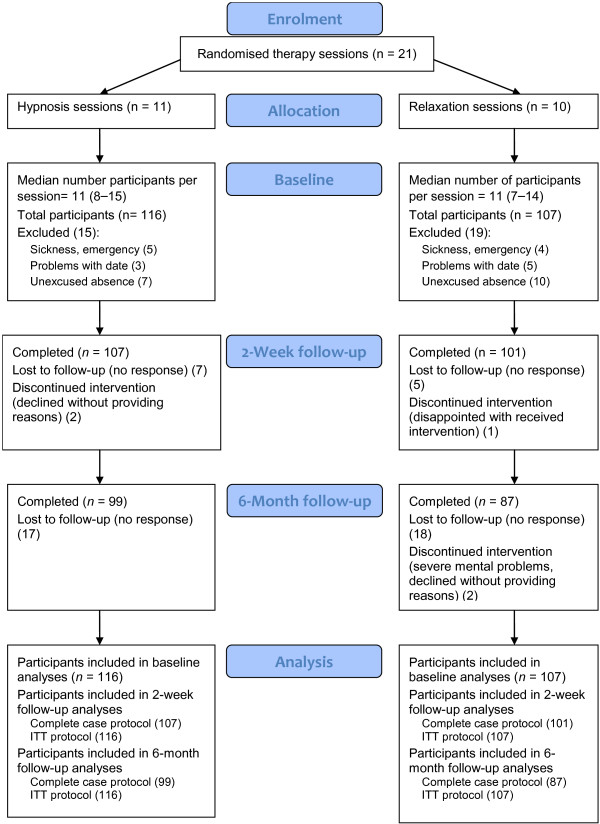
Participant flow through the trial.

Figure 1 shows the flow of the participants throughout the trial.

#### Recruitment

The participants were recruited constantly, starting mid-April 2011 and ending at the beginning of February 2012. Follow-up interviews took place continuously two weeks and six months following the therapy dates. Recruitment ended when we reached our intended sample size.

### Data preparation

Data imputation was undertaken in PASW 18.0 (IBM Corp., Armonk NY) for baseline variables with missing values. None of these variables had more than 9% missing values. Data were imputed through the linear regression method. Scale means for the FTND, self-efficacy, PCS-12, MCS-12, the MNWS, adverse events, BAI, and BDI-V were calculated.

### Baseline data

Table 1 shows characteristics of the sample. The relaxation group was significantly more educated compared to the hypnosis group which, in turn, showed higher FTND values and a slightly higher mean on the BAI than the relaxation group.

**Table 1:** Baseline characteristics of the hypnosis and the relaxation group and of the total sample

**Variable**	**Hypnosis (*n *** **= 116)**				**Relaxation (*n *** **= 107)**				**Total (*N *** **= 223)**				**Test**
	***n***	***%***	***M***	***SD***	***n***	***%***	***M***	***SD***	***n***	***%***	***M***	***SD***	***t, X***^***2***^
Demographics													
Female	52	44.8			53	49.5			105	47.1			0.50
Age	116		38.0	13.2	107		36.9	10.1	223		37.5	11.8	−0.71
Swiss	102	87.9			90	84.1			192	86.1			0.68
Civil status													2.04
Single	49	42.2			51	47.7			100	44.8			
Married	45	38.8			43	40.2			88	39.5			
Divorced/separated	22	19.0			13	12.1			35	15.7			
Education													11.22*
Basic education	13	11.2			4	3.7			17	7.6			
Apprenticeship	56	48.3			40	37.4			96	43.0			
Maturity	24	20.7			24	22.4			48	21.5			
University	23	19.8			39	36.4			62	27.8			
Smoking-related variables													
FTND	115		5.0	2.2	107		4.4	2.0	222		4.7	2.1	−2.13*
Self-efficacy	115		3.3	0.9	106		3.3	0.8	221		3.3	0.9	−0.43
Cotinine (ng/mL)	115		336.6	119.3	107		326.3	124.6	222		331.6	121.7	−0.63
Age at first cigarette	93		16.2	3.3	85		17.2	3.6	178		16.7	3.5	1.89
Smoking cohabitants	11	24.4			16	38.1			27	31.0			1.89
Cessation attempts													
Number	37		3.3	3.4	43		3.7	5.2	80		3.5	4.5	0.37
NRT	38	40.9			34	40.0			72	40.4			0.01
Alternative therapies	18	19.4			16	18.8			34	19.1			0.01
Physical health													
PCS-12	116		51.5	6.5	106		52.7	5.6	222		52.1	6.1	−1.56
Lung disease (life)	11	10.1			8	8.2			19	9.2			0.23
Heart disease (life)	6	5.5			3	3.1			9	4.3			0.74
Cancer (life)	4	3.7			1	1.0			5	2.4			1.54
Mental health													
MCS-12	116		50.0	8.4	106		50.1	7.5	222		50.1	7.9	−0.10
BDI	116		0.9	0.7	107		0.9	0.7	223		0.9	0.7	−0.95
BAI	116		0.4	0.4	106		0.3	0.3	222		0.4	0.4	−2.23*
Substance consumption													
Alcohol (30 days)	116		7.2	6.6	106		8.6	6.7	222		7.9	6.7	1.59
Alcohol (7 days)	116		1.8	1.8	106		2.2	1.6	222		2.0	1.7	1.61
Cannabis (life)	66	56.9			73	68.9			139	62.6			3.39
Cocaine (life)	13	11.2			13	12.3			26	11.7			0.06

*Note. M*, Mean; *SD*, Standard deviation.

*p ≤ 0.05. This table was created on the basis of the intention-to-treat dataset.

### Intervention and retention at follow-ups and delay of follow-ups

We were able to reach 92.2% (*n =* 107) of participants from the hypnosis group and 94.4% (*n =* 101) of participants from the relaxation group for the first follow-up. The difference in retention between the groups was not significant (*X*^*2*^*=* 0.41, *df =* 1, *p =* .52). For the second follow-up, we reached 85.3% (*n =* 99) from the hypnosis group and 81.3% (*n* = 87) from the relaxation group; again, this difference was not significant (*X*^*2*^*=* 0.655, *df =* 1, *p =* .42). The difference between the groups regarding the delay between the target and the effective follow-up date was neither significant at the two-week (*t* = 0.32, *df* = 193, *p* = .75), nor at the six-month follow-up (*t =* 1.37, *df =* 163, *p =* .17).

### Return rate of salivettes and biological validation of nicotine abstinence

All except one self-reported non-smoker returned the salivette. The one participant who did not return the salivette was counted as “*smoker*”. Biochemical analyses confirmed the nicotine abstinence, as defined by a value of <5 ng/mL, of 36 self-reported non-smokers, but three showed slightly higher values than those typically observed in occasional smokers. For our evaluation, these participants were counted as smokers.

### Outcomes and estimation

#### Primary outcome

At the time of the second follow-up, 17 (14.7%, 95% *CI* [0.08, 0.21]) of the participants in the hypnosis group and 19 (17.8%, 95% *CI* [0.10, 0.25]) in the relaxation group reported nicotine abstinence for the previous 30 days. The effect of the intervention was not significant (Table 2). Unadjusted analysis (without controlling for variables showing baseline group differences) led to a similar result (*OR =* 0.80, *SE =* 0.29, *t =* −0.62, 95% *CI* [0.37, 1.72], *p =* .54), as well as CC analysis (*OR =* 0.84, *SE =* 0.37, *t =* −0.40, 95% *CI* [0.34, 2.09], *p =* .69).

**Table 2:** Logistic regression model predicting 30-day point prevalence smoking abstinence at the six-month follow-up

**Predictor**	***OR***	**95% *CIs***	***SE***	***t***	***p***
Intervention (0 = relaxation, 1 = hypnosis)	0.86	[0.36, 2.06]	0.36	−0.35	.73
FTND	0.89	[0.72, 1.12]	0.09	−1.03	.32
BAI	1.21	[0.45, 3.26]	0.57	0.41	.69
Education					
Apprenticeship vs. elementary school	1.20	[0.20, 7.27]	1.03	0.21	.84
Maturity vs. elementary school	2.18	[0.31, 14.96]	2.01	0.85	.41
University vs. elementary school	1.42	[0.24, 8.37]	1.21	0.41	.68
(Constant term)	0.22	[0.0, 1.54]	0.20	−1.62	.12

Note. *OR*, Odds ratio; *CI*, Confidence interval; *SE*, Standard error.

#### Secondary outcomes

At the two-week follow-up, 38 (33.3%) of the participants in the hypnosis group and 26 (24.5%) of those in the relaxation group reported smoking abstinence in the previous seven days. The effect of the intervention was not significant (*OR =* 1.73, *SE =* 0.60, 95% *CI* [0.84, 3.57], *t =* 1.58, *p =* .13).

Two weeks after the intervention, the mean number of cigarettes smoked in the previous seven days was similar in both groups (Table 3). Linear regression showed that the effect of the intervention on the number of daily cigarettes smoked was not significant (*B =* .38, *β* = .03, *SE =* .94, *t =* 0.40, 95% *CI* [−1.58, 2.33], *p =* .69, *d* = 0.07, 95% *CI*(*d*) [−0.26, 0.40], *ICC* hypnosis = .00, relaxation = .00). Six months after the intervention, both groups showed higher cigarette consumption compared to the two-week follow-up (hypnosis *M* = 13.6, *SD* = 8.6, relaxation *M* = 14.3, *SD* = 7.3). The type of intervention did not significantly predict the number of daily cigarettes smoked at the six-month follow-up (*b =* −0.75, *β =* −0.05, *SE =* 1.27, *t =* −.59, 95% *CI* [−3.39, 1.89], *p =* .56, *d* = −0.09, 95% *CI(d)* [−0.42, 0.24], *ICC* hypnosis = .03, relaxation = .00).

**Table 3:** Means (M) and standard deviations (SD) for secondary outcomes at the two-week follow-up for the two intervention groups

**Secondary outcome**	**Hypnosis**		**Relaxation**	
	***M***	***SD***	***M***	***SD***
Daily cigarettes (previous 7 days)	9.40	7.70	8.90	7.00
MNWS	0.60	0.63	0.63	0.55
Smoking abstinence self-efficacy	4.31	0.67	4.22	0.80
Adverse events	1.22	0.30	1.19	0.24
Headaches	1.30	0.61	1.33	0.67
Dry mouth	1.41	0.78	1.32	0.67
Nausea and vomiting	1.06	0.28	1.08	0.34
Taste disorders	1.14	0.40	1.11	0.38
Visual problems	1.19	0.55	1.10	0.39
Abdominal pain	1.23	0.57	1.22	0.52
Constipation	1.30	0.65	1.21	0.52
Skin rash	1.13	0.42	1.11	0.43
Itch	1.18	0.58	1.18	0.48
Abnormal dreams	1.34	0.76	1.26	0.68
Other symptoms	1.13	0.51	1.20	0.61

The hypnosis group members showed a lower MNWS score than that of the relaxation group members. Linear regression analysis revealed that the intervention predicted the MNWS score, although the effect was very small (*B =* −.36, *β =* −.31, *SE =* .14, *t =* −2.54, 95% *CI* [−0.66, -0.06], *p =* .02, *d* = −0.05, 95% *CI(d)* [−0.58, 0.48], *ICC* hypnosis = .00, relaxation = .42). Members of both intervention groups showed similar self-efficacy (Table 3), and the intervention type had no effect on self-efficacy (*B =* .37, *β =* .37, *SE =* .24, *t =* 1.55, 95% *CI* [−0.13, 0.86], *p =* .14, *d* = 0.12, 95% *CI(d)* = [−0.41, 0.65], *ICC* hypnosis = .33, relaxation = .00).

The adverse event index, as assessed at the two-week follow-up, showed very similar means for the two intervention groups, and linear regression analyses confirmed the absence of an intervention effect (*B =* −.00, *β =* −.02, *SE =* .04, *t =* −0.24, 95% *CI* [−0.08, 0.07], *p =* .81, *d* = 0.11, 95% *CI(d)* [−0.17, 0.39], *ICC* hypnosis = .01, relaxation = .00).

### Self-reported use of other cessation aids and use of the CD

Of those participants who were abstinent at the six-month follow-up, one in the hypnosis group and five in the relaxation group had used other cessation methods between the therapy session and the six-month follow-up. The hypnosis group had used the CD, on average, 1.01 (*SD =* 4.32) times during the previous 30 days, and the relaxation group had used it, on average, 0.32 (*SD* = 1.54) times. This difference was not significant (*t* = 1.43, *df* = 115.86, *SE* = 0.48, *p* = .15, 95% *CI* [0.26, 1.64], *d* = 0.21, 95% *CI(d)* [0.09, 0.50], *ICC* hypnosis = .00, relaxation = .00).

## Discussion

This is the first study to investigate the efficacy of a single session of group hypnosis for smoking cessation in a large sample of smokers willing to quit. According to our findings, group hypnosis was not more effective for smoking cessation than group relaxation.

The abstinence rates resulting from our trial are comparable, despite figuring at the lower end, to other studies of a single session of group hypnosis (18.5-25% abstinence) [15,18,25]. Two studies reported higher abstinence rates of 45–50% [26,50]. Our abstinence rates are superior to the previously reported 7–8% success rate without cessation aids, which might be explained by our participants’ strong quit intentions [52], experiencing social support [53-56], having contact with a therapist, undergoing the psycho-educational part, or receiving suggestions in a relaxed state.

Both relaxation and hypnosis were associated with few adverse events two weeks after the intervention, and there were no group differences. Hypnosis seemed superior to relaxation alone with regard to attenuating withdrawal symptoms after smoking cessation. Psychologically, hypnosis did not lead to enhanced smoking abstinence self-efficacy compared to relaxation.

Our large sample size supports the generalizability of our findings to populations with a similar socio-demographical and cultural background and strong intentions to quit. On the negative side, generalizability might be lowered through the fact that in our study, the same therapist conducted all sessions, which does not allow quantifying therapist and intervention effects.

Our trial presents some limitations that need to be addressed. We debriefed our participants about their study condition because we wanted to prevent them from speculating about their condition and behaving according to their speculation. According to some participants, they were disappointed not to have received their desired intervention, which could have lowered their motivation to stay abstinent in the follow-up period. In retrospect, it would have been more elegant to leave our participants blinded until the second follow-up, while assessing their guess regarding their condition and their expectations of that condition.

The absence of between-group effects could lead to the idea that the participants in the hypnosis condition were in no different state compared to the participants in the relaxation condition. We were not able to check whether induction of hypnosis was successful as there was no self-report instrument with sufficient discriminant validity to differentiate between hypnosis and relaxation. Future research should deal with the question of how easily applicable and economic manipulation checks in hypnotherapy studies could be conducted.

## Conclusions

In conclusion, our study indicates that attending a single smoking cessation session containing a psycho-educational part and relaxation in a group of highly motivated smokers yields success rates of 15–18%. Hypnosis does not seem to have an effect beyond the non-specific effects of therapist contact, social support and relaxation in fostering smoking abstinence.

## Competing interests

The authors declare that they have no competing interests.

## Authors’ contributions

MDS coordinated the study, performed the statistical data analysis and wrote the manuscript. SH gave substantial input into data analysis and critically reviewed the manuscript. MS developed the study design. All authors contributed to and have approved the final manuscript.

## Pre-publication history

The pre-publication history for this paper can be accessed here:

http://www.biomedcentral.com/1471-2458/13/1227/prepub
